# I Am Not Just a Nurse: The Need for a Boundaried Ethic of Care in the Context of Prolific Relationality

**DOI:** 10.1007/s10551-022-05246-3

**Published:** 2022-09-10

**Authors:** Wee Chan Au, Siân Stephens

**Affiliations:** 1 Newcastle University Business School, Newcastle University, Newcastle upon Tyne, UK; 2 Business School, Middlesex University, London, UK

**Keywords:** Ethics of care, Nurses, Care at work

## Abstract

The Ethics of Care (EoC) theory has been widely applied in the field of management, and there is a growing consensus that it is important to recognise the value and practice of care in the workplace. In this paper, we consider the implications of the EoC at work, and in particular the risks unboundaried care demands may pose to employees who encounter unmanageable ‘calls to care’. We present findings from interviews with 27 nurses in Malaysia, which suggest that the demand to care at work, in addition to demands made in the non-work sphere, may be unmanageable. We argue for a more boundaried approach to the EoC at work with a view to ensuring that in valuing care we do not over-burden the carer.

## Introduction

Once a relatively niche feminist theory of philosophy and psychology, the Ethics of Care (EoC) has now found its place in mainstream management theory, particularly with reference to Human Resources Management (e.g. Dale, 2012), Leadership (e.g. Nicholson & Kurucz, 2019) and CSR (e.g. Spence, 2016). The EoC draws on observations of the moral development of women and girls to offer an ethical framework within which one is obliged to respond to the naturally occurring call to care elicited by an encounter with a needful other. The theory’s increasing academic application to leadership, work and employment is a timely reflection of the shift towards a more feminised workplace, where there are now more women than ever in paid work (Ortiz-Ospina et al., 2018) and in positions of leadership (Grant Thornton, 2021).

The EoC is a well-established perspective in disciplines that are more overtly care-related, such as informal care-work (Tuyisenge et al., 2020; Whitmore et al., 2015), social work (Lloyd, 2006; Meagher & Parton, 2004; Parton, 2003) and, in particular, nursing. The EoC has been a central concern of the field of nursing for many years (e.g. Botes, 2000; Crowley, 1994; Green, 2012; Myhrvold, 2006; Vanlaere & Gastmans, 2011); and discussions of the value and applicability of the approach have also evolved into a more critical consideration of the theory in nursing (e.g. Allmark, 1995; Kuhse, 1997; Paley, 2002). In the field of business and management, there is a growing body of work which evidences and advocates for a more caring approach in the workplace (Elley-Brown & Pringle, 2021; Lawrence & Maitlis, 2012; Paillé et al., 2016; Vijayasingham et al., 2018), demonstrating the relevance of the approach in a variety of professional context.

Applying the EoC to the workplace allows us to notice the relationality among colleagues and customers and provides a language with which we can account for the call to care that such relationality entails. The EoC also provides normative guidance, an account of what moral pre-act consciousness would entail, and in so doing, allows us to observe failures of morality, where the call to care is not fostered. There is considerable appeal in this; intuitively and experientially, we know that when the attitude of caring is found absent in the those we encounter, whether at work or at home, the experience is jarring and upsetting. However, we also note that heeding the caring imperative is a significant moral demand; it is manageable within a relatively small sphere such as the family because the boundaries are clear and the limitations reasonable. However, it is less manageable when we are called to care through endless encounters like call centre work (Witt et al., 2004), or nursing (Kim, 2020). In this paper we refer to such contexts as situations of ‘prolific relationality’.

Broad critiques of the theory in this vein relate to the implications of an approach where ‘one must always care’ and the anxiety associated with the pervading, unanswerable question ‘have we done everything our idea of caring demands of us?’ (Koehn, 1998, p. 23). The EoC theory acknowledges the reality that the distinction between the public and private sphere is, to a great extent, artificial. However, to date little has been offered in the way of advice on how to reject the unmanageable demands on one’s care which proliferate when these boundaries are blurred. The EoC offers an invaluable insight into how our experiences of care weigh into our ethical decision-making, and the theory facilitates the identification of demands for our care and an understanding of how we respond (e.g. when we feel compelled to act when met with need). It is argued here that to better realise the utility of the theory, consideration must be given to the implications of the approach when applied to all aspects of one’s life, including work. In particular, we assert with others (e.g. Koehn, 1998; Kuhse, 1997) that while there is immense value in identifying and understanding the call to care, the moral weight added to the caring attitude or impulse may make encounters with a needful other unmanageable when one is placed in a context of prolific relationality. While earlier EoC theorists point to the natural limitation of the demands on our care offered by our relationality (i.e. the ‘I must’ is only triggered on when one is in a close encounter with another), it is here suggested that these natural limitations cannot be relied on to ensure manageable circles of caring when we extend our consideration of ethical care to the workplace. Therefore, we seek a better understanding of the challenges of the call to care in a context of prolific relationality and of how these challenges are managed, to consider a more practical *boundaried* (as opposed to bounded) EoC, whereby the natural limits assumed by early theories are supplemented with more synthetic boundaries imposed by the one-caring. This led us to our research question, “how do women nurses, as professional caretakers and traditional domestic caretaker (as mother, wife and daughter), experience and manoeuvre the boundaries between their caring obligations in the work and nonwork domains?”.

With this in mind, we have analysed interviews with 27 nurses in a Malaysian hospital, applying the EoC theory to gain greater insight into the range of experiences of those who bear a heavy burden of caring at work, in pursuit of both theory development and a deeper understanding of care at work. The interviews provided insight into the rich diversity of caring acts inherent to such an explicitly caring profession and the diversity of care roles our interviewees performed as wives, mothers, friends, colleagues and women. As can be seen in the data discussed below, the experiences are, as one would expect, quite varied. All of our interviewees drew from a plethora of caring experiences, as the carer and as the cared-for, and a complex picture has emerged from the data, where the nurses navigate constantly evolving caring responsibilities at home and at work. This balancing act is fraught with difficulties and inconsistencies, but not without its rewards. From the interview data, we can identify multiple ‘circles of caring’ (groups of people for which the individual feels a caring responsibility), which can at times pull the nurse in painfully different directions, and at other times lead to the fulfilment of a highly compatible personal and professional identity.

The EoC allows us to notice and articulate how and why one might feel pulled in different directions when encountering many people in need, whom we are in a position to help. The application of the EoC to the workplace, particularly in environments where there is great need, reveals that there is potentially limitless ethical demand within the EoC framework (Tronto, 1993). Each of us has many overlapping circles of caring for whom we feel some degree of caring responsibility, beyond the more obvious circle of our immediate family and including our colleagues, clients, patients, service users, managers, employees etc. The naturally occurring ethical obligation to care for these groups more than others does not resolve the issue of how to balance our care for these groups, and it is these experiences of conflict which are reflected in our data.

In the next section, we present our theoretical framework, followed by a discussion of our methodology, data collection and analysis. Our findings are then presented and discussed, with consideration of implications for practice and future research.

## Literature Review and Theoretical Framework

### The Ethics of Care at Work

The origins of the Ethic of Care lie in a descriptive account of the psychological development of morality of girls and women (Gilligan, 1982), but as an ‘ethic’ (as opposed to an exercise of descriptive psychology), it is a set of moral principles (Held, 2006).

EoC theorists adopt a relational ontology, by which is meant ‘The ethics of care… characteristically sees persons as relational and interdependent, morally and epistemologically’ (Held, 2006, p.13). This can be contrasted with what might be termed an ontology of singularity, within which the fundamental nature of being can only be considered at the individual level, and all other aspects of existence (such as one’s positional relationality with the world and with others in the world) are secondary.

The Ethic of Care invites us to consider the normative implications of a relational ontology, and in so doing entails both a consideration of what is morality, and what it is to be moral. The EoC theorists do not seek conclusions which facilitate moral judgements of actions, but consideration of the morality of the ‘pre-act consciousness of the one-caring’ (Noddings, 1986, p. 28). The theory is not intended to identify right or wrong actions, but to identify the moral nature of ones encounter with the other, with the assertation that the ethical ‘pre-act consciousness’ is the moral imperative to care when one encounters the need of another (what Noddings calls the ‘I must’). In this way, the development of the theory has facilitated the elucidation of the subjective experience of an encounter with a needful other, as well as ascribing the turning to, as opposed to turning from, this need, a moral weight.

It is acknowledged that one will encounter situations where one *should* feel the call to care but does not, perhaps because the would-be cared-for is difficult to like, or because the one-caring is overwhelmed. In such instances, it is argued that, to appeal to what is ‘good’, one must call upon the moral imperative to care even where this is not felt naturally. ‘If we do not care naturally, we must call upon our capacity for ethical caring…The imperative in relation is categorical’ (Noddings, 1986, p. 47).

Where we refer to ‘care’ in this paper, we are referring to the moral pre-act consciousness of responding to the need of another person, and where we refer to the ‘performance of care’, we are referring to actions and behaviour associated with caring, arising either from natural caring or from the more effortful summoning of the ‘I must’ described by Noddings and associated with what is termed ‘ethical caring’ (1995, p.15). While such performance might, within other ethical frameworks, be considered ‘emotional labour’, it would not be characterised as such within an EoC. Within a framework where ‘values of trust, solidarity, mutual concern and empathetic responsiveness have (ethical) priority (Held, 2006, p.15), such ethical engagement would not be a suppression of one’s own naturally occurring emotional state to better align with a patriarchal capitalist system, but an engagement with one’s natural caring impulses.

It is argued that the human experience of moral behaviour is one which is derived from our best understanding of what it is to be cared for—usually, or perhaps ideally, based on our own memories of having been the recipients of care (Noddings, 2013). These memories are triggered and call us to action when presented with one in need. We are obliged to care where we can, but not where we cannot—i.e. we are not obliged to care for those with whom we have no relationship at all, or for those who need what we cannot provide, and we are not required to care for everyone equally. The boundaries of what is ethically required of us are thereby drawn along the lines of what it is possible for us to do, and we can identify to whom we have an obligation to respond by considering who’s situation it is within our power to change (Noddings, 1986).

The EoC is a revolutionary re-conception of ethics-in-practice. It has strong descriptive power; it speaks true to anyone who has ever felt more moved by the plight of those closest than those removed, and allows for a moral position where one is satisfied by the wellbeing of those who are close, alongside knowledge that there is suffering elsewhere. The EoC recognizes not only that care happens, but also that care is vital to the ongoing existence of the species and as such is a noble—and ethical—undertaking (Held, 1995). In ascribing moral value to the practice of care as it arises from the caring impulse, the theory elevates behaviour traditionally associated with girls and women. Care, which is by definition relational (one can care only about, or for—there is no care without a subject (Tronto, 1993)) has often been ‘relegated’ to the domestic, and therefore the private, sphere. This association of care with the domestic sphere compounds the denigration of care; care has been un-valued both because it is women’s work and because it takes place in the private home (which is in turn un-valued because it is the woman’s domain). The EoC elevates the status of care and, in so doing, paves the way for the valuation of those who’s morality is rooted in their caring.

The theory allows for the valuation of caring in the home, but it also encourages us to put aside the private–public distinction and to recognise, value and practice care wherever care is called for. As such, the theory can and has been applied to many fields of human social existence (Clement, 1996; Held, 2006) as there are many other contexts where one is faced with demands for one’s care. In considering the implications of the EoC, it becomes clear that we exist within a complex web of caring relationships, including many which are not of one’s own choosing (Held, 2006). The work context is one in which we are likely to encounter prolific relationality, confronted as we are with colleagues, clients, customers and suppliers, and it has been observed that the way in which we now think about experiences of work may lend itself to an EoC lens (Liedtka, 1996; White, 1992). The EoC approach has been applied to explore how firms engage with their stakeholders (Burton & Dunn, 1996; Elley-Brown & Pringle, 2021; Spence, 2016; Wicks et al., 1994), human resource management (Dale, 2012; Magrizos & Roumpi, 2020; Mariappanadar, 2012) and development (Armitage, 2018; McGuire et al., 2021), as well as leadership (Gabriel, 2015; Nicholson & Kurucz, 2019). It has been well established that care does and (in at least some circumstances) should occur in the workplace, and the value of the EoC approach for understanding the experiences of work and management is evident. However, there has been little consideration of the implications of an EoC at work for those doing the caring and little consideration of if, how and where to impose boundaries on one’s caring in the workplace (Antoni et al., 2020). The need for such exploration is relevant to any application of the theory, but it is particularly pressing when the theory is applied at work, as work is a context where the lines regrading to whom one owes one’s care may be blurry at best, and the risk of unmanageable care demands is high.

### Critical Perspectives of the Ethics of Care

Discussions regarding the EoC in the workplace have been ongoing in more overtly caring disciplines such as social work (Lloyd, 2006; Meagher & Parton, 2004; Parton, 2003) and nursing (Allmark, 1995; Nortvedt et al., 2011) for some time. In contexts such as these, professionals are involved in daily decision-making about allocating and prioritising their care. As such, the EoC approach has proven particularly fruitful, allowing for recognition and explication of how professional carers experience the ethical dimension of their caring work. In addition to a thoughtful application of the theory in the field, there have been robust critical responses, although none to our knowledge which specifically calls for greater consideration of how and where to apply boundaries to care at work *within* an EoC framework.

Broad critiques of the theory in the nursing and social work fields draw attention to the vagaries of identifying all care as at least potentially ethical and the moral problems inherent in such a position (Allmark, 1995; Kuhse, 1997; Rudnick, 2001). More discipline-specific critique revolves around what is seen as an unhelpful over-emphasis on the nurse-as-carer at the expense of their moral authority. It is argued that requiring nurses to have profound personal connections with their patients is asking both too much (as an exhausting and impossible endeavour) and too little (failing to recognise the value of their practical skills) (Kuhse, 1997) while perpetuating the subservient role of nurses in relation to doctors in what has been referred to as a Nietzchean slave morality (Paley, 2002). Such critiques point to the risks of a moral requierment of care-at-work (in any workplace), which may add a further burden of responsibility to employees, and in particular those employees for whom the expectations of and opportunity to care will be higher; either because of their gender, or their profession, or both (as in the case of women who are nurses).

While the possibility of overwhelming and competing care demand is acknowledged within the body of EoC literature, little advice is offered as to how this can be met and overcome. It is suggested that while ‘sometimes, the conflict cannot be resolved and must simply be lived (Noddings, 1986, p. 55), in general, our caring obligations will be bounded, with naturally occurring limitations, as we are called to care ‘only’ to those in and linked to our ‘inner circles’ (Noddings, 1986, p. 86). However, when the EoC is applied to work, the circle of caring is extensive, particularly for those in professions where one encounters a high number of people in need. In such contexts, we cannot rely on our caring being ‘bounded’ in the sense of an externally imposed limitation, as the nature of the work ensures that no such limitation is likely. It is argued here that we should instead consider the value of care which is ‘boundaried’, i.e. limited by boundaries imposed by the one-caring, particularly in the context of the relationality encountered in virtue of one’s work.

To date, literature that addresses the application of an EoC at the workplace tends to either present an EoC in the workplace as either an ideal (e.g. Elley-Brown & Pringle, 2021; Spence, 2016; Wicks et al., 1994) or, as in some of the nursing and social work literature (e.g. Allmark, 1995; Paley, 2002), reject it entirely. Research is required to better understand the implications for those involved in professions where an EoC is likely to be observed. Such research would help demonstrate the benefit of an EoC lens in identifying caring-at-work, and allow us to consider the need for a boundaried EoC when applied in the workplace.

The research presented in this paper addresses the experiences of nurses, and as such, builds on the extant research which considers the EoC in the context of nursing. However, the interviews and the findings do not focus exclusively on the specific caring demands the interviewees experienced as nurses but on more universal experiences of care for and from their colleagues, supervisors and the institutions within which they work, as well as the non-work sphere. The findings presented below are of value in any professional context where one is likely to encounter the call to care—arguably almost any professional context at all.

## Method

### Participants

The nurses in our sample are employed in a public hospital setting where a full range of public healthcare services is provided. The majority, 20 of 27 nurses, were required to work in shift patterns except those working in the specialized clinics and support departments (e.g. central sterile services department). The average age of our interviewees was 36, and all interviewees were married except one who was divorced. The average number of children for our interviewees was 2.6, and this number includes the three who did not have children. All of our interviewees were women. The career span of the interviewees working as a nurse ranged from 4 to 27 years, and all but six participants (who had worked only at their current health site) had worked in more than two health sites, either clinics or hospitals. The interviews covered the participants’ whole career journey and were not just limited to their experiences in their current workplace. Appendix Iprovides a description of the sample.

### Procedures

#### Abduction as a Methodological Approach

**Fig. 1:**
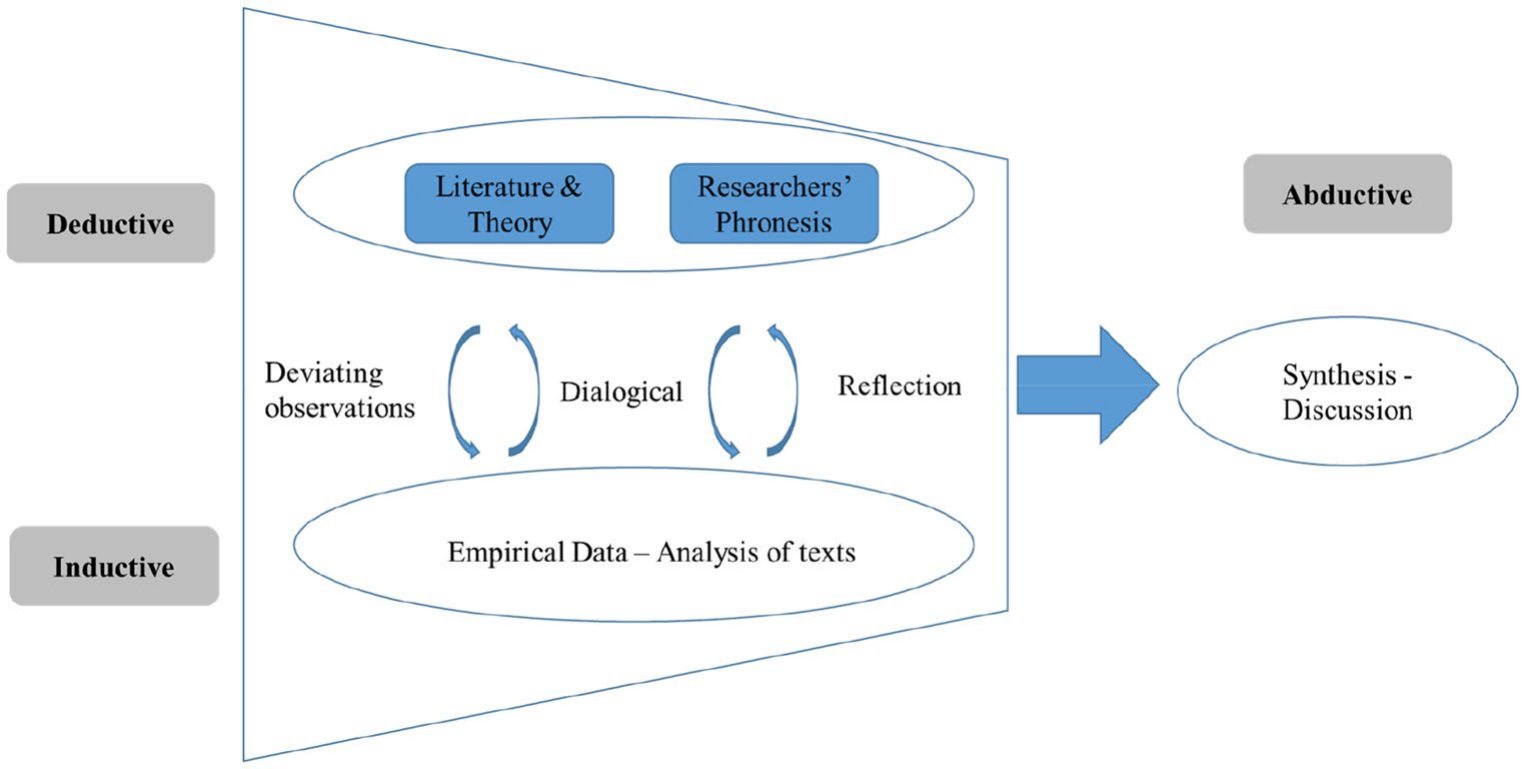
Overview of abductive research process. Adapted from Dubois and Gadde (2002) and Kovács and Spens (2005)

We followed a common prescription for abductive approach (Dubois & Gadde, 2002, 2014; Reichertz, 2010). An abductive approach is particularly useful in providing meaningful explanations of new discoveries, as it captures both theoretical concepts and empirical evidences (Dubois & Gadde, 2002; Hurley et al., 2021). Approaching the data with an EoC lens, we paid close attention to the lived caring experiences of nurses, with a view to developing an understanding of how nurses meet the extensive care demands being made of them at home and at work. While the description of the procedure is linear for readability purposes, the process was highly iterative, moving between and among data, relevant theory and literature, and emerging themes (Dubois & Gadde, 2002). In the abductive approach, the continuous interplay between theory and empirical observation is stressed, and the approach facilitated theory development rather than theory generation or confirmation (Dubois & Gadde, 2002, 2014; Reichertz, 2010). Induction and deduction function as a springboard for the abductive leap, which enables scientific synthesis (Thorkildsen et al., 2013). In other words, the abductive approach moves back and forth between deductive understanding of prior theoretical knowledge and inductive interpretation of the empirical data. See Fig. 1 for a visual presentation of the abductive research process. Such an approach has allowed us to consider the implications of the EoC at work and to observe the unanticipated empirical findings, on which we draw to advocate for a boundaried EoC.

##### Empirical Data Collection

In order to investigate the ways in which those in positions of prolific relationality navigate extensive care demands, we analysed the lived experiences of 27 nurses from a public hospital in Malaysia. The data were collected through in-depth semi-structured interviews, which were conducted to explore how nurses maneuver between different care roles in both work and nonwork domains.

This study is a part of a larger project that examined the health and wellbeing of nurses. The Medical Research and Ethics Committee, Ministry of Health Malaysia (NMRR-18-3129-44821) and the Monash University Human Research Ethics Committee (1814) approved the study. The recruitment of interviewees took place through a call for volunteers by the hospital to their nurses. Interviews were scheduled with nurses who indicated a willingness to participate and provided telephone numbers at the end of the questionnaire of the bigger project. All interviews were digitally recorded. Interviews lasted 45 to 70 min, with the average interview lasting 50 min. Most interviews were conducted at the hospital’s meeting space, except two held in cafés during the interviewees’ day-off. All interviews were transcribed verbatim. The first author, an academic based in Malaysia, conducted all of the interviews. The collected data was in Malay, the official language in Malaysia. We used professional transcribing and translation services to transcribe and translate the interviews. The first author checked all of the transcripts against the audio recordings to ensure the accuracy and consistency of translation, reflecting the participants' original meaning and tone.

##### Empirical Data Analysis

Our data analysis focused on understanding how women nurses, as professional caretakers and traditional domestic caretakers (as mother, wife and daughter), experience and maneuver the boundaries between their caring obligations in the work and nonwork domains. We were inspired by Gioia et al. (2013)’s data structure approach to help us structure and present the inductive data analysis. This is widely adopted in qualitative studies in presenting the data structure (e.g. Byrne et al., 2019; Hennekam et al., 2020). Inductive data analysis, complemented by the constant comparative method (Glaser & Strauss, 1967; Miles & Huberman, 1994), proceeded in four stages.

The first stage aimed at creating meaningful units of analysis where the transcripts were read line by line to identify relevant references used by the participants. Through this open coding procedure, we coded meaningful units using simple phrases close to the language used by the interviewees to reflect the first-order categories. During this step, recurring ideas emerged from the interview data. We aimed to categorize interview data into first-order categories that gave voice to the nurses about their lived experiences in managing the care roles in work and nonwork spheres. This stage revealed the expectations that the nurses should care (e.g. extended circles of care and the expectations to care in all spheres of life; the expectations to care outside of work hours and beyond professional duties), the experiences of nurses’ in managing care in work and nonwork domains (e.g. the competing demands in both domains, nurses rely on spouse support and outsource certain domestic care works), as well as the influence of care from employers on nurse’s experiences (e.g. being cared for by the employer and the adjustments made at the workplace to accommodate competing care demands). We constantly compared meaningful units within and across transcripts to ensure similar experiences were grouped together. The constant comparison also took place within a category to refine the label of each category. In sum, 17 first-order categories of experiences concerning nurses’ care roles were identified.

Stage two involved sorting these ideas into broader sub-categories. We engaged in axial coding to consolidate first-order categories into broader sub-categories with the theoretical explanations of nurses’ EoC. Further groupings of similar sub-categories into a narrower set of themes occurred at stage three. At this stage, we developed seven second-order themes that reveal narrower relationships between sub-categories that serve as the foundation of theoretical interpretations in the next step. Again, we used constant comparisons to delineate and differentiate identified second-order themes.

**Fig. 2:**
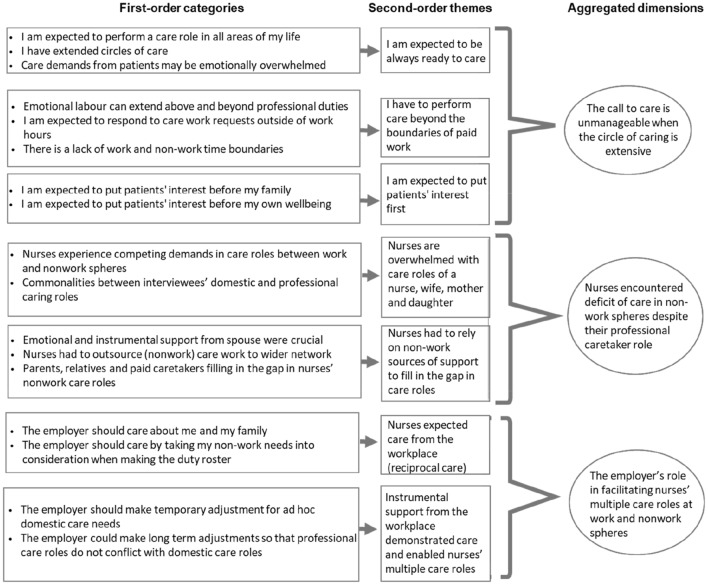
Overview of coding and data structure

Stage four involved theoretical incorporation, where the second-order themes were further aggregated into high-order aggregated dimensions. We combined related themes into the aggregated dimension and sought insights into the relationships between dimensions. At this stage, we developed aggregated dimensions via an EoC lens: we elevate the experiences of care, highlighting the unmanageable demands of the call to care in a context of prolific relationality (capturing the expectations to be always ready to care, care beyond work boundary and the expectation to put patient’s interest first). We also synthesized the multiple ways nurses cope with the deficit of care in nonwork spheres, and finally, we identified that nurses are also longing to be cared for. This dimension captured the experiences of the nurses as the cared-for and highlighted the instrumental support from the employer as a demonstration of care. Figure 2 presents our resulting data structure, which provides a graphical representation of the relationships among the first-order categories, second-order themes, and aggregated theoretical dimensions (Gioia et al., 2013).

## Findings

Three themes emerged from the analysis; two of which address the role of our interviewees as the one-caring, while the third reveals the experiences of nurses as the cared-for. The emergent themes are: (1) The call to care is unmanageable when the circle of caring is extensive, (2) Nurses encountered deficit of care in non-work spheres despite their professional caretaker role, and (3) The employer’s role in facilitating nurses’ multiple care roles at work and nonwork spheres. Our themes have been identified from categories that were mentioned by at least nine participants, which is approximately one-third of the interviewees. In the subsequent paragraphs, we illustrate these three prevailing themes that emerged from the analysis of interview transcripts. We discuss these findings supported by quotes from the participants. (refer to Appendix II for additional quotes for illustration).

### Theme 1: The Call to Care is Unmanageable When the Circle of Caring is Extensive

While we approached the analysis with an EoC lens, seeking to better understand the way in which our respondents experience care as an ethical practice, unmanageable nature of the call to care when working in a context of prolific relationality and extensive need was a commonly identified theme. The EoC theory allows us to notice and understand that where there are caring (or non-caring) acts, there is also a ‘pre-act consciousness’, and maintains that this is the locus of morality. While the nurses often refer to care in quite a literal sense, they are also referring to their own pre-act caring attitude and impulse, and commenting on the difficulties they encounter when such attitudes and impulses are absent. The findings discussed in this theme highlight the way in which the pre-act disposition to care can be undermined when the call to care is pervasive, as well as the challenges of managing and prioritising competing moral demands.

#### I Am Expected to Be Always Ready to Care

All participants referred to a constant expectation of care within their professional role as nurse. The nature of their work is that it deals with life and death, and with such high stakes the nurses had difficulty with the boundless nature of the call to care from their work. H19 explained the challenges this posed for her, and her inability to cope with the incessant care demands, “You can’t say no. You might have your off day planned out, but you must respond when [care] duty calls. You must do so without question. I was about 40 [years old] and I couldn’t handle it any more”. In addition to the expectation that they be available to work in a physical sense, participants expressed frustration at being expected to care throughout their work shift. Such expectations from the patients and their families, even from their co-workers, place nurses under tremendous stress. H23 said,

They [the patients’ families] will come during visiting hours and complain about whatever they see. […] during visiting hours, I spend less time with the patient. I let the family spend more time with the patient […] I use that time for documentation, sometimes at the counter. Then they complain we are not doing our job. They said, “the nurses write most of the time; they don’t help the patient.” That’s what I feel is the most challenging. They come after I do my work and say I don’t do my work.

While such an expectation is perhaps not surprising from patients and their families, the expectation to constantly care also came from the nurses’ extended circles of care. The interviewees explained that their care roles and responsibilities are not confined to the hospital’s boundaries; their professional caretaker role means that extended family and neighbours identify the nurses as carers and hold expectations that this role be extended. Some of the nurses were also the main caretaker of their extended family members. These community expectations may reflect the collectivist culture in Malaysia, where loyal membership is stressed, and one takes responsibility for fellow members of their group, be that a family, extended family or extended relationships (Hofstede Insights, 2021). While most participants were happy to use their caretaking knowledge and experiences to help where they could, some admitted that such expectations placed an additional burden on them. H2 shared:

Because we know each other, people from the [same] village will ask, "Can you please help me keep an eye on my child [in the ward]?" or help them keep an eye on their relatives... So I feel a huge burden. […] Once, my brother-in-law asked me to take a look at his mother...I told him I was working, but I will go after work […] I feel like I cannot fully carry the responsibility they gave me because I have other responsibilities at work...I have my patients...I have other responsibilities. So I can't cope.

Most of the participants described the expectations of their care as constant, and the care demands from patients could be overwhelming. As professionals in a role where they encounter a significant amount of need, they are expected to be always ready to care and serve, but some respondents expressed difficulty coping when their natural impulse was not one of caring but one of ‘anxiety and anger’. As H18 shared,

Our patients are challenging at times. We are of the intention to help, but they wouldn’t cooperate. […] we want to help them, but they would throw a temper. So we must remain calm all the time. Even when the patients scold us, we must deal with them with a smile on our faces. Only after they left, we can show our true emotions to our friends [colleagues]. It is my responsibility to control the negative emotions. [...] If I am anxious and angry all the time, high blood pressure or other side-effects might come knocking on my door. Aren’t they frightening?

#### I have to Perform Care Beyond the Boundaries of Paid Work

The nurse’s accounts reflect a porous boundary between work and home. They describe expectations that the call to care they experience at work be available outside work hours, as the need of their patients and colleagues is continuous. As the hospital operates 24/7, it is common to receive notifications, instructions and requests from colleagues and supervisors on duty anytime throughout the day. Accordingly, the nurses are expected to attend phone calls and WhatsApp messages from the hospital even off duty. H10 shared,

Sometimes our head nurse wants urgent updates, so we have to attend to her. […] Say you are on vacation, then suddenly you hear notifications from your phone (Imitating mobile phone ringtone) “Please update. If not, may get a legal case.” It can be disturbing at times. “Please update why the baby did not go through SPO2?” I received that when I was on holiday. I had to reply.

If necessary, they would be asked to return to the hospital. This happens when there are emergency cases during their on-call shift or to take over from colleagues who had to take emergency or medical leave. H2 shared the frustration of how her nonwork spheres were interrupted by her care role at the hospital:

There is no choice but to answer to those [phone calls and WhatsApp] even after working hours. Sometimes I feel mad because I have arrived home, but I have to turn back to the hospital; […] If I'm on-call, I have to accept it...but if I'm not on-call, I feel annoyed. I have to reply whether or not I want it, because we should always be ready to help. I had given feedback and asked why the management does things this way. They just said, "We are ready to serve.” What to do? We need to uphold that slogan.

Our interviewees recognised that the negative emotions resulting from the demands of their formal care responsibilities might be carried over to their nonwork spheres. The nurses’ nonwork domain is inevitably affected by their professional care role, and some report diverting the negative emotions from the hospital to their family members. According to H07:

Nurses are constantly being humiliated. I often get frustrated about that, and at times I would direct my frustration towards my husband. When I am back from work, I will grumble at my husband, and he will comfort me. “It is the nature of your work.” “Let it go, okay?”

#### I Am Expected to Put Patients’ Interest First

Interviewees describe the way in which their managers and employers expect that they prioritize their care-at-work, even when they face competing care demands from work (e.g. colleagues in need) and nonwork domains. In some cases, the participants felt that they had been unable to support and care for their families when they were desperately needed. The nurses offer accounts of instances where they remained at the health site to take care of the patients and rely on others to meet their families’ care needs. According to the nurses’ account, decisions around prioritisation are not made on (solely) pragmatic or practical grounds but as a caring response to the need with which they are confronted. H24 recounted her experience during a natural disaster in the region:

There was a flood, a big flood. […] I’ve been taught discipline. So I came to work on a lorry. […] and we got stranded. We couldn’t return home. We worked for 24 hours. The helicopter kept coming, transporting patients. I didn’t know what had happened to my house. I wasn’t able to contact my family. I was crying over here while pushing patients [...] I didn’t know if anything happened to my mother. The entire area was flooded badly. Whenever I think about home, I would complain, “why did I come to work?” But then I also thought, “there’s no one else. It would be worse if I didn’t come”.

The nurses also prioritize their professional care work over their own health and wellbeing, and again this was not done for fear of being penalised, but as the result of a caring consideration of need and their own ability to respond. In many cases, the nurses continued to report to work even when they were not feeling well, and they explained that this was because it is not possible to delay the care work at the hospital – patient care is not work that can be caught up on after taking a few days off, so if they take sick leave, another nurse has to cover for them. Accordingly, most of the nurses refrained themselves from taking sick leave so that the care work in the ward was not interrupted and so as not to make additional demands of their colleagues. H25 stated:

I have to work [even when I am not well]. If the ward is busy, I have to put aside my own health, as long as it’s not detrimental. I feel bad for my colleague. […] So if you have a headache, you swallow Paracetamol and get back to work. It happens pretty often.

The participants described how the nurse’s uniform gave them strength and called their responsibility for professional care roles, which help them push through even when they feel sick. In putting on the uniform the interviewee was assuming the role of professional carer, and communicating “nonverbal, conscious statement that nurses have the skills and knowledge to care for others” (Spragley & Francis, 2006, p. 58). The uniform allowed the wearer to give precedence to her professional call to care over any other emotions or obligations she may have been experiencing. H7 recalled her experience when she was pregnant:

I had hyperemesis and was admitted to the hospital four times. […] When I am in my uniform, I become strong but I become weak when I am not. In my uniform, I can work as normal [even when I am not well]. Throughout eight months of pregnancy, I conducted 21 home deliveries. […] Be it at work (health site), or when I am on call, I give my work my best because work is my priority. If I were to collapse, I would collapse after I finish my job. Treat others before myself. That is my principle.

### Theme 2: Nurses Encountered Deficit of Care in Nonwork Spheres Despite Their Professional Caretaker Role

Almost all the nurses indicated that they experienced a sense of deficiency in their caring at home as a result of their care-at-work, suggesting that they were not able to easily manage the competing calls to care of their prolific relationality. The hospital adheres to the labour laws and the governmental guidelines about hours of work, breaks, and the number of paid leaves. Other than nurses who work in the specialized clinics and support departments (e.g. central sterile services department), most participants are working on the 24-h scheduling in rotation. The schedule of the rotating shift works is: 7 am to 2 pm (morning shift); 2 pm to 9 pm (afternoon shift); and 9 pm to 7 am (night shift). They work on the allocated shift for a week before working another shift. Hence, working at night, weekend, and public holidays are common among the participants. Under normal circumstances, nurses only serve one shift within the 24-h scheduling. However, double-shift may occur when one is required to cover a colleague who is on emergency leave. Occasionally, one may be “on-call”, where the nurse needs to serve as a backup workforce to attend to emergency cases if there is a staff shortage. They will be compensated accordingly for work beyond their usual work commitment. Based on the nurses’ accounts of the competing care demands from work and nonwork domains (refer to Fig. 2), we derived two sub-themes described below.

#### Nurses are Overwhelmed with Care Roles of a Nurse, Wife, Mother and Daughter

The nurses encountered competing care demands from the hospital and home. In most cases, it was felt that the interviewees’ care as mother, wife and daughter deferred to their care-at-work. As healthcare workers in a public hospital, their work routines are erratic; a conventional 8am to 5 pm work schedule was not the norm for most interviewees. In addition, the need for health care services 365 days requires nurses to be on duty on weekends, school holidays and public holidays. Hence, being able to take days off during school holidays and public holidays was considered a luxury. The constraints of their professional schedule impacted their ability to perform care roles at home as they wish and the requirement to work night shifts, be on-call schedule, cover for colleagues etc. was an added burden on the nurses’ deficit of care at home. The sense of being pulled in two directions meant for some that they were unable to focus their attention on any one demand. As H22 stated:

I feel like I spend a lot of time in the hospital compared to being at home. Sometimes I work in the morning [shift]. After that, there’s CNE (Continuous Nursing Education^1^) in the evening. It’s supposed to finish at 3.30 pm, but sometimes it finishes at 4 pm […] that disrupts my routine, it takes time out of my day... it intrudes on the time I get to spend with my family […] I can’t even focus with the CNE because I will be thinking about my children, who will pick them up and things like that.

Respondents were mindful of the limited staffing resources of the hospital and the impact of unplanned absences on colleagues (who would be required to come into work on short notice) and, for this reason, were reluctant to take un-planned time off. Nonetheless, the nurses also acknowledged the irony of providing care all day at work while relying on family members or babysitters to care for their own children, and we are reminded of the unmanageable obligations identified in Theme 1. When confronted with needs at home and the needs of colleagues and patients at work, the caring disposition of the nurses is placed under considerable strain. While they are mentally prepared for such sacrifice, such moments can still give rise to a sense of guilt. As H8 explained:

As a nurse who is also a mother, perseverance is crucial. While our job is to treat a patient, it is ironic that we have to report to work even when our children fall sick. We have to put our children aside. […] instinct tells me that my children’s sickness is manageable and that my babysitter can manage them, I will still report to work. All I can do is to remain strong and pray for my children’s safety. There are [many] considerations to be made before I take a leave. Will there be enough staffs at work? Will there be a lot of patients? Will the ward be busy? […] Nonetheless, when we were trained in nursing [school], we were trained to think a certain way. It is planted in our minds that we must be ready to sacrifice our family for work.

#### Nurses had to Rely on Nonwork Sources of Support to Fill in the Gap in Care Roles

While the nurses themselves encountered prolific relationality and extended circles of caring due to their work, they relied quite heavily on the care of the more discrete, traditional circle of caring of their immediate family, as well as on paid childcare services. Almost all participants stressed the importance of spousal support in terms of psychological and physical support to help them overcome the deficit of care at home. Most nurses highlighted the role of their husband in taking care of children whenever they work at night shifts and when there is ad hoc work duty, excepting those whose husbands were often away from home for work. Sixteen of the interviewees explicitly described the supportive behaviour of their husbands. For example, when talking about her husband, H1said,

I'm thankful that my husband understands. We've been married for 13 years, and from the start, he never stopped me from working night shifts. "It's okay, you just go to work," he said. When our kids were still young, he was the one taking care of them at night. He works office hours, so he would take care of the kids at night, and in the day, he would send [them] to day-care [center]. My husband is very good at handling them. When I said “"Mama is going to work during Raya (Eid holiday)”, my kids would surely say, "oh no! mama, why don't you take leave?" Then my husband would handle the situation. "It's alright, Papa will be here, Papa will bring you here and there". When I said I have to take care of patients, even when our kid was sick, he would say "it's okay, you go to work. You prepare the medication; I will give it to the kid later". I feel blessed.

Understanding and supportive parents and parents-in-law, and even siblings were an important source of support for these nurses in addressing the care deficit at home. Nurses who were staying with or near their extended family members relied on their family members for both housework and childcare. Sixteen of the participants identified the extended family as the caregivers for their children. According to the interviewees, even when extended family members are not the regular caregivers, they serve as important backup support on an ad hoc basis whenever the babysitter or the husband is not available. H17 shared,

I live near my mother. When both my husband and I are at work, she can look after the kids. I work on shifts, sometimes I will be at home in the morning and will only leave in the afternoon. So, the kids will have to stay at my mother’s place for a while. […] Living near my mother and siblings makes it easier […] if my mother is not available, my siblings can look after the kids for me.

The participants shared the challenges they encountered when sourcing external support. As H16 shared, “Babysitters don’t like babysitting a nurse’s baby because we work on shifts. Normally babysitters wouldn’t accept a night job.” Therefore, nurses who do not access family support for childcare had to outsource the care work to paid childcare services, including babysitters, childcare centres, kindergarten, and afterschool daycare centres.

As H21, who is a Malay, explained how sending her children to Chinese-medium school is a way to ease her burden:

We sent them to a babysitter when they were young. My husband had always been [posted to an] outstation. He would only be at home for two to three days a week. So, I had to handle the kids [by] myself. It was a tough time for me. I sent them to kindergarten as soon as they were two [years old] […] They all go to Chinese schools. I am sure you know Chinese schools have many [after school] activities. That makes my life easier.

### Theme 3: The Employer’s Role in Facilitating Nurses’ Multiple Care Roles at Work and Nonwork Spheres

Our interviewees also reflected on their experiences as the cared-for, highlighting the important roles of employers in facilitating nurses’ multiple care roles at work and nonwork domains. When understood within the EoC framework, the recipience of care is a vital ingredient in one’s moral development towards being an ethical provider of care; ‘when the attitude of the one-caring bespeaks caring, the cared-for glows, grows stronger, and feels not so much that he has been given something as that something has been added to him’ (Noddings, 1986, p. 20). In theme three (refer to Fig. 2), the nurses expressed a strong desire to be cared for by the hospital, their supervisors, and their co-workers, as well as the belief that such provision would help them to better navigate the caring demands described in Theme Two. The important role of workplace support for taking care of the nurses’ interest was manifested through two sub-themes we describe below.

#### Nurses Expected Care from The Workplace (Reciprocal Care)

Most interviewees explicitly expressed expectations of care from their workplace, including from colleagues, supervisors and the hospital as an institution. Beyond work-related support, the nurses feel a strong need for their nonwork lives to be understood and for their employers to care about them and their family members at a personal level. The expectations of care from supervisors towards their subordinates were clearly articulated in the nurses’ accounts. For example, H22 highlighted the importance of caring gestures from her supervisor:

It’s important [that the manager gets to know me] because I’m not just working [as a nurse], but I have children […] It’s not just about work. She [the Sister] should know me for who I am, what my husband works as, how many children I have, who takes care of them. When problems arise, for instance, I have a problem with my babysitter. When I share it with her, she’ll understand. If I have a problem and want to change shifts, she would already know my situation. That’s why I said she needs to know me on a deeper level.

Almost all nurses interviewed felt that their supervisors’ fairness and transparency in the arrangement of the duty roster was or would be an example of care, a finding which offers both support and challenge to the principles of the EoC approach. In order to fulfil their care demands at home, the nurses with children are competing to take days off during school holidays and public holidays so that they can spend more time with their families. Faced with a sense of competition for a limited resource, interviewees expressed a preference for fairness and transparency, not partiality. When talking about her experience in requesting days off, H8 said:

Conflict arises typically during school holidays. Everyone will fight for leave so that they can go on vacation. Some Sisters are fair; some are not. The Sisters should not be prejudiced. It is not right to let the same person be constantly on leave. Sometimes we see the same person on long leaves. Some Sisters allow that. Maybe they like the person a lot, or that person licked their boots well. Some sisters will only allow short leaves, like a 2-day leave for a person they are not fond of.

#### Instrumental Support from the Workplace Demonstrated Care and Enabled Nurses’ Multiple Care Roles

In addition to the need for both care and fairness in the allocation of shifts, our interviewees expressed a need for instrumental support to help them cope with ad hoc nonwork care demands or long-term adjustments to suit their nonwork needs. While most nurses avoided sick leave, there were unforeseen circumstances where emergency leave was required. As H14 described,

When our children fall sick, and need an emergency leave, she (my supervisor) is considerate of our situation. She will look for our partners [replacement] instead of us [looking for replacement] when we are on leave.

The participants also shared their appreciation towards the supervisors and the hospital for making long term work adjustments, such as transfer of health sites or departments, to accommodate their nonwork care roles. Examples include the gestures of being cared for by the workplace to allow them to perform care roles at home, including children, elderly, and special needs families. As H9 recounted,

At times he [the son] would wear his shoes wrongly. He cannot differentiate between B and D even though he is 11 years old now. He requires constant monitoring. Realizing that, I had to request for an office hour job so that I could monitor his studies at night. If I work in the afternoon [shift], I will reach home at 9 pm. By then, he will be too tired to do anything. If I work at night [shift], I will not have time for him […] The supervisor was empathetic. She listened to me and immediately approved my request upon understanding my difficulty.

## Discussion

The final stage of the adductive approach involves the scientific synthesis, referring to the discussion in conventional research work (Thorkildsen et al., 2013). In this stage, the research team engaged with existing interpreted knowledge in the process of adductive reasoning. The findings presented here describe both the lived experience of the EoC and the need for a boundaried EoC. Our interviewees are clearly caring people – they speak of their care for those they encounter at work and in their community spontaneously and in detail. Previous research describes how women experience social expectations to provide care at both home and work (Brue, 2018; Dutta, 2018), but social or cultural expectations do not wholly account for the experiences offered by our interviewees. While there is some suggestion our interviewees prioritise care-at-work because they are required to do so by their employer, the more prevalent account is one whereby the prioritisation is based on an assessment of need and the individuals’ ability to respond to the need. The accounts provided by our interviewees illustrate well the experience of a lived EoC when the obligations of such an ethic are applied in all spheres of one’s life. In particular, they describe experiences of care being called on because it is ‘the right thing to do’, such as H2, who responded to an unwelcome message to come back to work with the attitude ‘I have to reply because we should always be ready to help’. Thus the imperative of relationality described by Noddings (1986) is demonstrated, where the call of ‘I must’ is heeded, even if it is not easy or uncomplicated to do so. Our findings also add insight into the lived experiences of the cared-for, and it is evident that the nurses themselves benefit from being the subjects of care, e.g. from their supervisors, to help them better navigate the competing demands in their own lives. The nurses described the benefit of ensuring their supervisor was familiar with, and therefore caring about, the nurse’s personal circumstances. These findings add to evidence that the quality of the relationship between supervisor and subordinate is a form of socioemotional resource that is beneficial for employees’ work-family outcomes (Major & Morganson, 2011; Morganson et al., 2017), as well as evidence of the practice and value of care in the workplace.

However, our findings offer new insight into the implications of an ethic of care in a context of prolific relationality. The call to care at work and at home placed burdensome and often conflicting obligations on the interviewees. While the web of support offered by spouses, extended family and babysitters adds further evidence of relational care, it also illustrates the way in which the mothers and husbands could more easily heed the call of the ‘I must’ in the relatively discrete circle of caring of their own family, to be contrasted with the multiple calls to care being encountered by our interviewees. The account of an interviewee who was asked by a neighbour to provide particular care to a family member illustrates the consequences of an unboundaried EoC well: “I feel like I cannot carry the responsibility they gave me fully because I have other responsibilities at work… I have my patients…I have other responsibilities. So I can't cope” (Interviewee H2).

Extant research shows that work that demands too much of us can cause real harm, (e.g. Kayaalp et al., 2020; Kramer & Son, 2016), which is supported in our findings. For example, interviewee H18 describes high blood pressure resulting from suppressing anxiety and anger; Interviewee H7 explains that she works even when she is sick because “*If I were to collapse, I would collapse after I finish my job*”. Interviewee H25 explains that she will go to work when she is sick out of consideration for colleagues who would have to cover if she took time off. The data does not suggest that our interviewees push themselves in this way because the hospital demands it; rather, they do it because they care. They also care about their families, and one would hope that they care about and for themselves too. The data show us that where there are no clear limits to the care applied at work, these competing care demands can easily become unmanageable.

The EoC approach is rooted in an attempt to better establish the realities of ethics experience and demarcate a more realistic map of our ethical obligations. However, it is not clear from the initial formulations of the theory how best to manage what ethics requires of us in contexts of prolific relationality, or if there are spheres within which our obligations differ (Clement, 1996). The findings presented in this paper offer an insight into the way in which the naturally occurring limits to our caring obligations may not obtain, as well as evidence that nonetheless some limits are necessary. Therefore, it is concluded that a boundaried ethic of care is called for, within which it recognised that the calls to care one encounters at work may be responded to differently than those we encounter within more naturally occurring bonds. Such a conclusion is within the scope of the EoC, which is concerned in a significant way with the appropriate limitation of one’s ethical obligations. We move beyond what has already been theorised however in asserting that the one-caring be entitled to impose such limitation, rather than simply being subject to them.

Although the data were collected prior to the COVID-19 pandemic, the findings have a strong implication on the health and wellbeing of nurses given the stressors, strains, and needs for resilience and care among health care professionals during the global pandemic. Owing to pandemic fatigue, burnout and exhaustion, healthcare workers, including nurses worldwide, call to be cared for while coping with the pandemic's stress (The Star, 2021; The Sun Daily, 2021; UCA News, 2021). Nurses are already suffering from burnout due to the staff shortage; additional sources of stress during the pandemic have heightened the pressure on nurses in overlooking their nonwork care roles. During this period, many nurses isolate themselves from their families for an extended period to avoid transmission of the virus; they also bear the emotional labour to facilitate communication between the COVID-19 patients and their loved ones (Mehta et al., 2021; The Star, 2021). This exacerbates the pressure in balancing the care roles in both work and nonwork domains.

Our data are drawn from a professional context where the care demand at work is particularly apparent. However, the findings that our interviewees respond to need in the workplace as an ethical call to care and that they, therefore, struggle to manage competing care demands, can surely be applied in any context where one is confronted with extensive meetable needs. The extant literature applying the EoC theory to the workplace demonstrates that care is present in a wide range of work contexts, and anyone who has worked will recognise the inevitability of caring for colleagues, clients, customers, service-users or students. The findings presented here encourage us to consider the development of the EoC theory to include a clearer application of boundaries and a greater consideration of the realities of unmanageable care obligations.

We would suggest that relationality arising from paid work be considered as different in nature to that arising from naturally occurring bonds. If this were done, we may better realise the helpful, natural limitation of our obligation to care envisaged by early theorists. Applying a boundaried EoC to the workplace would allow us to identify and value care in the workplace, facilitating the elevation of care and those who perform care and ensuring that the care burden is not unseen and therefore unlimited. In moving away from a universal approach to ethics, we are in a better position to draw lines around where and when a call to care must be heeded. Whereas the theory in its original iterations allowed us to prioritise care based on the urgency of the need, our findings here suggest that there are some work contexts, e.g. those who work in healthcare, where the consequence of this would be to consistently prioritise care at work over care in the non-work sphere. If, however, the boundaries were drawn so that care arising solely from relationality at paid work was not permitted to compete with more naturally occurring care, a situation might arise where care is both highly valued and practicable.

## Appendix I

See Table 1.

**Table 1:** Participant characteristics

ID	Number of children	Marital status	Age	Work in Shifts	Career duration (years)	Number of served health sites
H01	2	Married	38	Yes	12	3
H02	3	Divorced	36	Yes	12	3
H03	1	Married	31	Yes	8	1
H04	2	Married	34	Yes	10	2
H05	0	Married	28	Yes	3	2
H06	0	Married	43	No	19	2
H07	2	Married	44	Yes	21	2
H08	2	Married	37	Yes	13	1
H09	2	Married	34	No	14	2
H10	2	Married	30	Yes	7	2
H11	2	Married	33	Yes	11	1
H12	3	Married	39	No	15	3
H13	4	Married	43	No	21	3
H14	2	Married	31	Yes	7	2
H15	3	Married	31	Yes	15	2
H16	3	Married	34	Yes	10	2
H17	3	Married	35	Yes	12	2
H18	0	Married	31	Yes	7	1
H19	4	Married	50	No	27	2
H20	3	Married	36	No	15	3
H21	4	Married	43	Yes	18	8
H22	3	Married	26	Yes	4	1
H23	3	Married	37	Yes	14	3
H24	4	Married	42	No	18	1
H25	4	Married	43	Yes	19	3
H26	4	Married	45	Yes	18	4
H27	4	Married	44	No	16	3

## Appendix II

See Table 2.

**Table 2:** Illustrative quotes for second-order themes from nurses

Second-order themes	Illustrative quotes
I am expected to be always ready to care	We are facing a lot of challenges […] we receive complaints that we lack professionalism at work… there is a gap [misunderstanding] between patients and nurses. We have been doing our jobs. The patients are ignorant about our workflow. Take sitting at the counter as an example. When, in fact, we are looking through their profiles, they would think otherwise. They would think that we are doing nothing or that we are chit-chatting. (H18) Each ward has 30 patients. There are only two or three working nurses. […] The patients always complain. But what they don’t know is that nurses are chasing down work all the time. That’s the burden. I feel pitiful of myself. To make things worse, the high-ranked specialist complains too. Once a medical officer commented, “Are the nurses here to do paperwork or here to take care of patients?’ This is the things that I’m sick of. If they are outsiders, it’s ok if they don’t understand, but… (H24)
I have to perform care beyond the boundaries of paid work	When I am at home, and if my neighbours have a fever or a sickness, they will come to me for my opinion. “If I have these symptoms, what should I do?” “Should I see a doctor?” Before they go to a clinic or a hospital, they come to me to check their blood pressure or whatnot. (H13) When I return home at 4 pm [after my afternoon shift], I will check my WhatsApp for any message addressed to me to see if anything is left behind during my shift…There might be an emergency [call/message midnight], my partners might need to take an emergency leave. I will keep an eye out for those situations where I might be asked to work. (H14) When I am back from work and get called back to work… that is the only thing that would upset him (the husband). He never complains about my work routine, but when I am back from work and get called back to work, he would [be upset], his mother too. (H19)
I am expected to put patients' interest first	I had to report to work at the flood center during the flood. Although my house was also affected, I didn’t get to sit around at the flood center like a flood victim. I had to work, check BP, check the temperature, give paracetamol, check baby and everything else. That's the most challenging (moment)… My husband was at home. He had to pay two persons to help carry our refrigerator, washing machine, furniture and put at an elevated place… At that time, I try to think that “okay, they are really in need of people to work”. But I still feel frustrated. I'm forced to…. I still have to work [even when my house was flooded]. (H01) Sometimes she (the mother) was sick for a week, but I couldn’t go home (at hometown). She said, ‘Oh, you only came home when I’m well.’ What can I do? I have a job. I can’t do anything. When the roster is out, I don’t want to disturb my colleagues. I visit her whenever I get annual leave. It’s far from here to my hometown, about seven hours (one way by driving). (H26) [When I am not well] So long as I have the energy, I will report to work. […] If I take the day off, everyone will be affected because changes must be made [to the duty roster]. Every time someone goes on leave, I feel the trouble, especially during night shifts. When I am done with my rotation finally deserve a break, I have to cover for someone who goes on MC, although the day is supposed to be my off day. For that reason, I always go to work even when I am sick. If I faint, they can always send me to the Emergency Unit. Haha. (H09) When I was pregnant, I had evening signals. When I was working nights, I would vomit, experience indigestion. So I attached a drip to my hand while I worked. I was with my drip when I was treating patients. I pushed the drip stand when I was treating patients. […] Because if I’m on MC, someone has to replace me. So I’ll be troubling other people. So even when I’m not feeling well, if I’m able to come to work, I’ll come to work. (H23)
Nurses are overwhelmed with care roles of a nurse, wife, mother and daughter	When they were younger, they often caught fever or other sicknesses. There should be some time for us to focus on our family, and I speak for everyone. My colleagues faced the same difficulty of requesting an EL (emergency leave). Some even brought their kids to work. They’d leave their kids in a room and check on them once in a while. This is depressing. Why is asking for an EL this difficult? We take care of strangers, but we can’t do the same for our family? (H07) My kids are always with our babysitter. I feel bad about having too little time to spend with my kids… There is more work in the hospital than at home. Also, I don’t get to be off duty always. If my husband is off duty, I will be working. If I am off duty, my husband will be working. So, if we were to travel, we would have to plan. It is difficult to find time for my family. (H04) It’s hard. My work ends in the ward, I won’t bring it back home. But for my responsibilities as a wife [and mother] […] When he [the husband] is at home, I have to focus on him. When I go home from work, I’ll pick up the children directly from the school. Then I’ll handle the children. In the evening and at night, I’ll focus on my husband and my children…. It’s tiring because I have to deal with everyone from morning until night. I have three children; I have to attend to all three [of them]. (H22) My family has to sacrifice a lot. The kids spend lesser time with me and spend a lot of time with the babysitter. If I am on-call, I can be called here any time after work. Sometimes I have to work following the on-call schedule. From working in the morning, if I am on-call at night, I have to work at night as well. So it’s my family that has to sacrifice a lot. Even when it comes to my husband, our vacation times are not in sync. For example, this upcoming school holiday, we’ll send the kids to my hometown because my leave is not approved. I will send them for a week or two, then bring them back here. (H23) Even though I don’t have kids, when I get home, I have to attend my mother’s needs. She would say she wants to go out or wants some something else. Even though I’m tired, I have to keep that aside and settle that one extra duty. That adds a little bit to my stress. (H24)
Nurses had to rely on non-work sources of support to fill in the gap in care roles	When I was with the surgical department, I never came home [if I am on call], so I have to…apologise to my husband, "please look after the kids"…because it's already late at night and I am not home. I will only be home at 2 pm the next day. After I finish work in the morning, I will sleep at the hospital and continue with my next shift straight away. Because I don't know what time I will finish [attend the case], it doesn't make sense for me to go home at 2,3am and then wake up at 5am to cook and go back to work. I won't have enough rest. So I have to get help from my husband. My husband has to understand too. If he doesn't, then it will be difficult. (H20) He [the husband] understands. My work is in shifts, morning, afternoon, or night (shift). If I work at night, he'll be the one who takes care of my child…He doesn't say it[anything], but he shows it [care]…Taking care of my child is important because it's exhausting when you're already tired from work. (H03) When my children were younger, I was at ABC. So I sent them to a babysitter. She reduced my burden when I had to be ‘on call’. On Saturdays and Sundays, we would visit the houses of postnatal patients. During that time, my husband was working in the army. We definitely needed a babysitter to take care of our children as they were all little. (H27) I am fortunate to have my mother-in-law. She took care of my children when they were young […] Because of her, my burden was lessened. […] For morning shifts, she would come the night before; For afternoon shifts, she would come in the morning. My husband is mostly available anyway. So, I am able to work excessive hours. […] It is truly a blessing to have it easier than the others… Inshallah, what a hassle it would be if I were to hire a babysitter. (H19)
Nurses expected care from the workplace (reciprocal care)	Last time when I was at ABC, there was this Sister. If our kids and husband were sick, she had never told us that we cannot take leave. She said "It's okay, if your husband, kids or family is sick…you [can] take EL. You don't have to come to work. […] If anything happens to your loved ones, I can't replace [them]" […] "If you can’t work, I will find someone to replace [you] […] We will do what we can" she said. "Work will never finish. Patients are patients; we do have to take care of them. But our family [fall ill], if you come to work, your heart will not be at peace, and you won't be able to give the best service to your patient. You will be thinking of your kids, your husband, your mother at home," she said. She is very motherly. (H01) The Sister [supervisor] is like an actual older sister to me. The matron would be my mother here. Sisters have to take care of their siblings, right, the younger siblings (subordinates)? Appropriately, of course. You have to be fair and love them all equally. Don’t show favouritism. And with the matron, she’s my mother. I have to listen to her instructions, like how I would be with my actual mother. (H24) Our Sister comes out with a request log [for leave]. If many requested leave on the same date, the Sister would have no choice but to prioritize those who need it the most rather than those who want it for recreation, like attending a wedding function or whatnot. Some needs the leave for emergencies. The Sister will prioritize those who need it because we can’t all go on leave… From the request log, we can count the number of staff who will be working, so we can plan when we want to go on leave. When our applications don’t get approved, we will sort them out. (H17)
Instrumental support from the workplace demonstrated care and enabled nurses’ multiple care roles	If I must leave to attend to a matter at home, she’d [the supervisor] be okay with it. She’d turn a blind eye and allow me to leave, as long as I come back to work after [I have settled the urgent matters]. She’d cover for me in the meantime. That happened quite often. (H04) In the beginning, my sister’s condition wasn’t severe. She had high blood pressure and diabetes. As time passed, she started to have mild strokes. It was then I requested a transfer. I only waited for two weeks as my sister couldn’t get up on her own. They understood my situation and approved my transfer quickly. Otherwise, I would have to wait for a long time. Because of my Sister, I got to come back to my hometown, and I am here to date. (H06) I transferred here [a department that works usual office hours] for my children. My husband had to work far from home and couldn’t look after the kids. I had to do everything myself. That was why I made the request, which was only approved three months later…. I need Fridays and Saturdays off to look after my children, to pick and send them, to watch them, or whatnot. It would be difficult to switch places with others should I work on shifts. Everyone has their personal problems to attend to. Not fair that I am the only one [goes on leave on weekends regularly], is it? Say I need to fetch my children every Saturday. I can’t be taking all Saturdays off, can I? What about others then? So, it is better to be here. (H19)

## Footnotes

1 Training provided by the hospital to improve quality of health care.
